# Antibiotic use in elderly patients in ambulatory care: A comparison between Hungary and Sweden

**DOI:** 10.3389/fphar.2022.1042418

**Published:** 2022-11-17

**Authors:** Ikhwan Yuda Kusuma, Maria Matuz, Réka Bordás, Maria Juhasz Haverinen, Muh. Akbar Bahar, Edit Hajdu, Ádám Visnyovszki, Roxána Ruzsa, Péter Doró, Zsófi Engi, Dezső Csupor, Ria Benko

**Affiliations:** ^1^ Institute of Clinical Pharmacy, University of Szeged, Szeged, Hungary; ^2^ Pharmacy Study Program, Universitas Harapan Bangsa, Purwokerto, Indonesia; ^3^ Albert Szent-Györgyi Health Centre, Central Pharmacy, University of Szeged, Szeged, Hungary; ^4^ Public Healthcare Services Committee, Stockholm, Sweden; ^5^ Department of Pharmacy, Faculty of Pharmacy, Universitas Hasanuddin, Makassar, Indonesia; ^6^ Albert Szent-Györgyi Health Centre, Department of Internal Medicine Infectiology Unit, University of Szeged, Szeged, Hungary; ^7^ Institute for Translational Medicine, Medical School, University of Pécs, Pécs, Hungary; ^8^ Albert Szent-Györgyi Health Centre, Emergency Department, University of Szeged, Szeged, Hungary

**Keywords:** drug utilization study, ambulatory care, antibacterials, elderly, cross national comparison, prescrptions/1000 inhabitants/year, public health, antibiotic stewardship

## Abstract

**Background:** The elderly use antibiotics frequently due to their increasing infection susceptibility. Given the high and increasing proportion of elderly in the population, their antibiotic use is substantial. *Objective:* This study aimed to compare antibiotic use in the elderly in the ambulatory care sector between Hungary and Sweden.

**Methods:** This retrospective, descriptive, cross-national, comparative study included antibacterial use data from the Hungarian National Health Insurance Fund and the Swedish eHealth Agency. Antibiotic use (anatomical therapeutical chemical: J01) was expressed as the number of prescriptions/1000 inhabitants/year or month and was further stratified by age and sex.

**Results:** Antibiotic exposure was higher in the Hungarian elderly population (649.8 prescriptions/1000 inhabitants/year) compared to its Swedish counterparts (545.0 prescriptions/1000 inhabitants/year). Hungary had a similar scale of antibacterial exposure across all elderly age subgroups, with different trends in males and females, while Sweden had a stepwise increase in antibiotic exposure by age in both sexes. The seasonal fluctuation was high in Hungary and reached a peak of 80.7 prescriptions/1000 inhabitants/month in January 2017, while even antibiotic use was detected throughout the year in Sweden. The pattern of antibiotic use in the elderly considerably differed between the two countries. Penicillin and beta-lactamase combinations, such as co-amoxiclav, were more frequently used in Hungary than in Sweden (19.08% vs 1.83% of corresponding total ambulatory antibiotic use). Likewise, quinolones were more commonly used in Hungary than in Sweden (34.53% vs. 9.98). The elderly in Sweden were mostly prescribed narrow spectra penicillins (26.71% vs. 0.29% in Hungary).

**Conclusion:** This cross-national comparison revealed important differences in all aspects of antibiotic use in the elderly between the two countries. The identical scale and pattern of antibiotic use cannot be anticipated due to the poorer health status of the Hungarian elderly population. However, the substantial differences indicate some room for improvement in the antibiotic prescription for the Hungarian elderly.

## Introduction

Antimicrobial resistance (AMR) implies a threat to global human health. Contributing factors of AMR include antibiotic overuse and misuse in hospital and ambulatory care settings (Ventola, 2015). Current demographic projections show an increasing elderly population in Europe. In 2019, proportion of the elderly population (≥65 years) proportion in Europe, Hungary, and Sweden was 31.4%, 29.3%, and 31.9%, respectively, of the total adult active (15–64 years) population, which is projected as 39.1%, 33.7%, and 34.4% by 2030, respectively (Eurostat, 2019).

The elderly population is at increased risk of many infectious diseases due to progressive functional decline of the immune system, commonly referred to as immunosenescence (Feehan et al., 2021). Age-related immune system changes affect innate and adaptive immune responses (Feehan et al., 2021). Research data on outpatient antibiotic use in the elderly remained scarce despite the growing population size of the elderly in Europe, and most studies focus on long-term care facilities (Raban et al., 2021). Comprehensive country-wide data on antibiotic use in the elderly in ambulatory care have only been published for a limited number of countries, including Denmark (Jensen et al., 2021), Norway (Blix et al., 2007), and the United States (Kabbani et al., 2018). Moreover, no cross-national comparison research has compared antibiotic use for the elderly in ambulatory care between European countries. Therefore, this study aimed to compare antibiotic use in the elderly in the ambulatory care sector in Hungary and Sweden.

## Methodology

### Study design and setting

This retrospective and descriptive cross-national comparative study collected data on antibacterial prescriptions dispensed at community pharmacies in Hungary and Sweden in 2017. Antibacterials were classified according to the anatomical therapeutical chemical (ATC) classification system defined by the World Health Organization (WHO), version 2022 (WHO, 2020). The use of systemic antibacterials (ATC: J01) was measured as prescriptions/1000 inhabitants/year or month. The elderly population (aged >65 years) of Hungary and Sweden in 2017 served as study populations for this study, including 1,828,226 elderly in Hungary and 1,976,857 elderly in Sweden (data derived from Eurostat). The two populations were further stratified into subgroups according to age (65–69 years, 70–74 years, 75–79 years, 80–85 years, and >85 years) and sex. Seasonal variation of antibiotic consumption was also assessed.

### Description of databases

Data on antibacterial use was obtained from the Hungarian National Health Insurance Fund and the Swedish eHealth Agency. Both the Hungarian and the Swedish national health insurance systems cover almost 100% of the population of each country. The database in Hungary contains records of all dispensed and reimbursed ambulatory care prescriptions issued by general practitioners (GPs), specialists, and dentists to ambulatory care patients, nursing home residents, and patients visiting private practices (e.g., gynecologists, dentists). The drug coverage is approximately 95% because non-reimbursed antibiotics are not included in the database.

The Swedish database contains data on all dispensed antibiotic prescriptions providing 100% drug coverage. All medications prescribed to outpatients (irrespective of reimbursement status) that are issued by GPs, specialists, dentists, patients visiting private practices, or nursing homes are included in this database.

### Statistics

Excel was used for the statistical analyses, and visualization was done by the R package (version 4.1.2).

### Ethical considerations

Ethical approval was not needed because aggregated data were collected for both countries.

## Results

### The scale of antibiotic use

The entire Hungarian population (approximately 9.8 million people) was dispensed 6,792,714 prescriptions of antibiotics in 2017, 17.5% of which were dispensed to the elderly. Concurrently, the entire Swedish population (approximately 10 million people) was dispensed 3,204,838 prescriptions of antibiotics, 33.6% of which were dispensed to the elderly. The antibiotic exposure was 649.8 prescriptions/1000 inhabitants/year in Hungarian and 545.0 prescriptions/1000 inhabitants/year in the Swedish elderly population.

**FIGURE 1:**
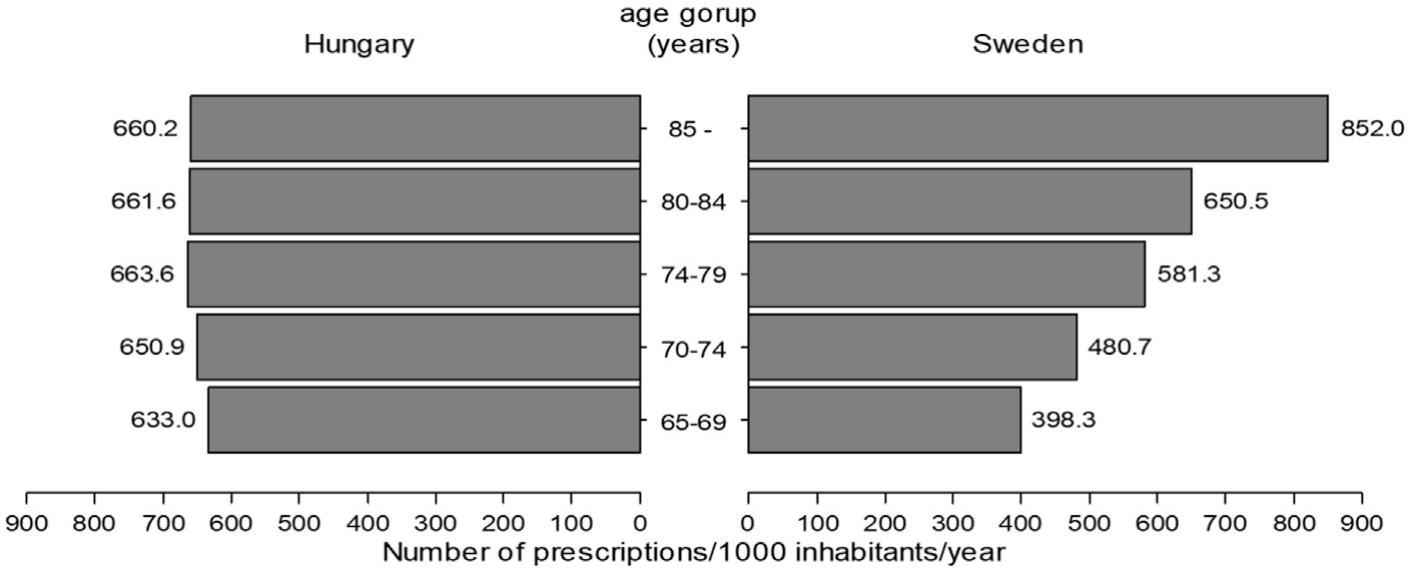
Antibacterial use in different elderly age subgroups in Hungary and Sweden (2017).

Figure 1 presents the level of antibiotic exposure across the elderly age subgroups. The antibacterial exposure of the Hungarian elderly population was similar across all age subgroups, while a stepwise increase was observed in antibacterial exposure by age subgroups (an increase from 398 [65–69 years old] to 852 (>85 years old) prescriptions/1000 inhabitants/year) in the Swedish elderly population.

### The pattern of antibiotic use

Table 1 shows the absolute and relative use of different antibacterial subgroups. Concerning the beta-lactam antibacterials, the penicillin group in Hungary was responsible for one-fifth of total ambulatory care antibiotic use in the elderly, and cephalosporins also had considerable use and share. In contrast, the penicillin group in Sweden was responsible for almost half of antibiotic use in the elderly, and marginal cephalosporin use was observed. The absolute and relative use of macrolides and fluoroquinolones were considerably higher in the Hungarian elderly population than in the Swedish counterparts, with an opposite pattern for tetracyclines and other antibacterials because their use was higher in the Swedish elderly (Table 1).

**TABLE 1:** Absolute and relative use of different antibiotic subgroups in the elderly population in Hungary and Sweden.

	Hungary	Sweden
J01A Tetracyclines	15.46 (2.38%)	52.84 (9.7%)
J01C Beta-lactam antibacterials, penicillins	141 (21.7%)	260.53 (47.81%)
J01CA Penicillins with extended spectrum	15.12 (2.33%)	105.03 (19.27%)
J01CE-CF Narrow-spectrum penicillins	1.90 (0.29%)	145.55 (26.71%)
J01CR Penicillin combinations, including beta-lactamase inhibitors	123.99 (19.08%)	9.96 (1.83%)
J01D Other beta-lactam antibacterials	75.45 (11.61%)	9.14 (1.68%)
J01DB First-generation cephalosporins	0.60 (0.09%)	8.79 (1.61%)
J01DC Second-generation cephalosporins	58.36 (8.98%)	0.01 (>0.01%)
J01DD Third-generation cephalosporins	16.49 (2.54%)	0.26 (0.05%)
J01E Sulfonamides and trimethoprim	36.18 (5.57%)	28.56 (5.24%)
J01EA Trimethoprim and derivatives	-	13.93 (2.56%)
J01EE Combinations of sulfonamides and trimethoprim, incl. derivatives	36.18 (5.57%)	14.63 (2.68%)
J01F Macrolides, lincosamides, and streptogramins	120.06 (18.48%)	32.41 (5.95%)
J01FA Macrolides	82.86 (12.75%)	8.41 (1.54%)
J01FF Lincosamides	37.20 (5.72%)	24.00 (4.4%)
J01M Quinolones	224.38 (34.53%)	54.41 (9.98%)
J01X Other antibacterials	36.17 (5.57%)	106.96 (19.63%)
J01XE Nitrofuran derivatives	0.02 (>0.01%)	57.17 (10.49%)
J01XX Other antibacterials (e.g., fosfomycin, methenamine)	36.12 (5.56%)	49.09 (9.01%)
Other	1.11 (0.17%)	0.11 (0.02%)
Total (J01)	649.81 (100%)	544.96 (100%)

Unit = Prescriptions/1000 inhabitants/year.

Table 2 shows the top ten list of antibacterials. Amoxicillin and clavulanic acid (co-amoxiclav) and two fluoroquinolones (levofloxacin and ciprofloxacin) covered almost half (46.6%) of the antibiotic use of the Hungarian elderly population in ambulatory care (Table 2), whereas 40% of all antibiotics used by the elderly population in ambulatory care constituted of the narrow-spectrum penicillin V, flucloxacillin, or pivmecillinam in Sweden. Nitrofurantoin use was almost absent in Hungary but constituted approximately 10.5% of the elderly antibiotic use in Sweden.

**TABLE 2:** The top ten list of antibacterials used in the elderly population in Hungary and Sweden (2017).

Hungary	Prescriptions/1000 inhabitants/year	Percentage	Sweden	Prescriptions/1000 inhabitants/year	Percentage
co-amoxiclav	123	18.95	phenoxymethylpenicillin	81.5	14.95
levofloxacin	95.8	14.75	pivmecillinam	72.3	13.27
ciprofloxacin	83.9	12.92	flucloxacillin	64.0	11.75
azitromycin	57.1	8.78	nitrofurantoin	57.2	10.49
cefuroxim	48.2	7.42	ciprofloxacin	52.8	9.68
clindamycin	37.2	5.72	methenamine	48.5	8.90
sulfamethoxazole/trimethoprim	36.2	5.57	doxycycline	47.9	8.80
fosfomycin	36.1	5.56	amoxicillin	32.7	6.00
norfloxacin	24.5	3.78	clindamycin	24.0	4.40
clarithromycin	23.3	3.59	SMX/TMP*	14.6	2.68

* SMX/TMP, sulfamethoxazole and trimethoprim.

#### Sex-specific antibiotic use

Overall, elderly females used more antibiotics than elderly males in Hungary and Sweden. Elderly females have been exposed to antibiotics at 668 prescriptions/1000 elderly females/year in Hungary, while elderly males at 620 prescriptions/1000 elderly males/year. Swedish elderly females were exposed to antibiotics at 618 prescriptions/1000 females/year, while elderly males at 460 prescriptions/1000 males/year in ambulatory care.

**FIGURE 2:**
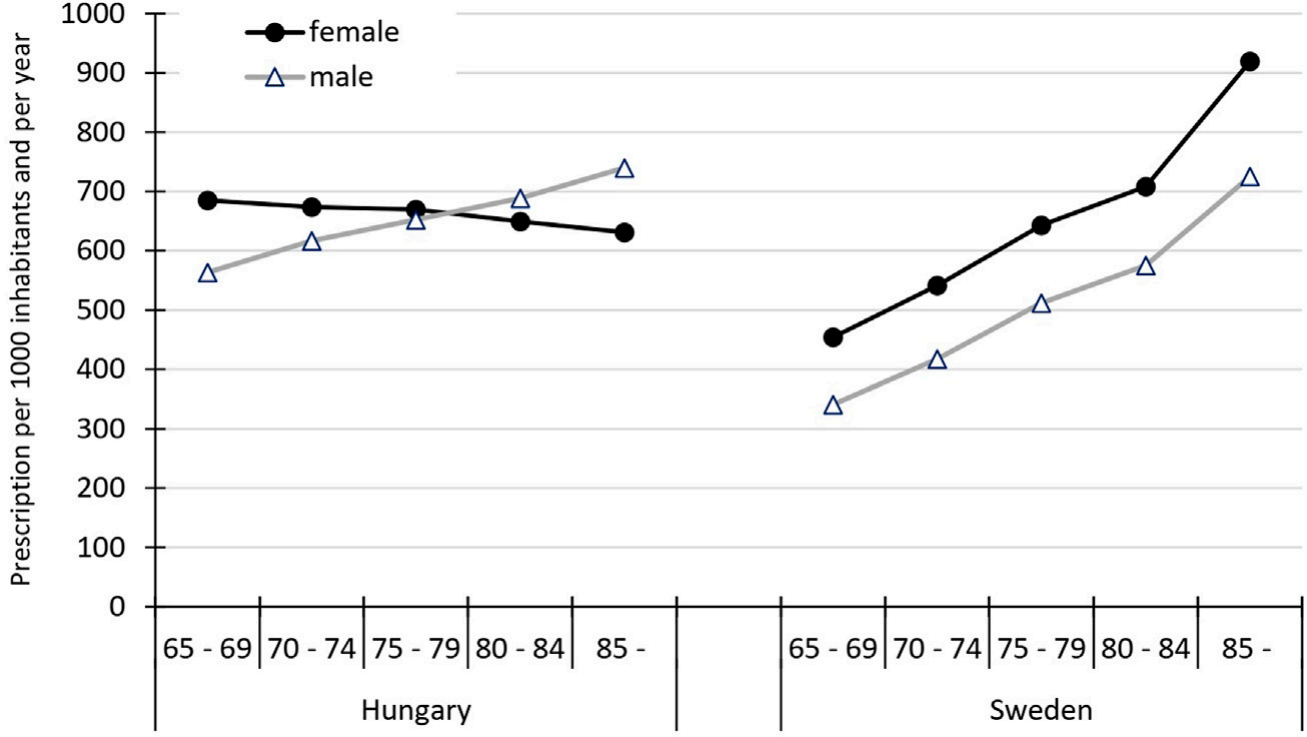
Sex-specific use of antibiotics in ambulatory care presented by age subgroups in the elderly population in Hungary and Sweden (2017).

However, the antibiotic exposure of the two sexes of the elderly population showed opposite trends in the age subgroup analysis in Hungary (Figure 2). Antibiotic use decreased from 685 prescriptions/1000 females/year (60–65 years old) to 631 prescriptions/1000 females/year (>85 years old) in Hungary. Conversely, the scale of antibiotic use in the Hungarian elderly male increased by age [from 563 prescriptions/1000 males/year (65–69 years old) to 739 prescriptions/1000 males/year (>85 years old)]. Both elderly females and males in Sweden were exposed to increasing amounts of antibiotics by increasing age (Figures 1, 2) and in all elderly subgroups Swedish females were exposed to more antibiotics than Swedish males).

#### Seasonal variation

**FIGURE 3:**
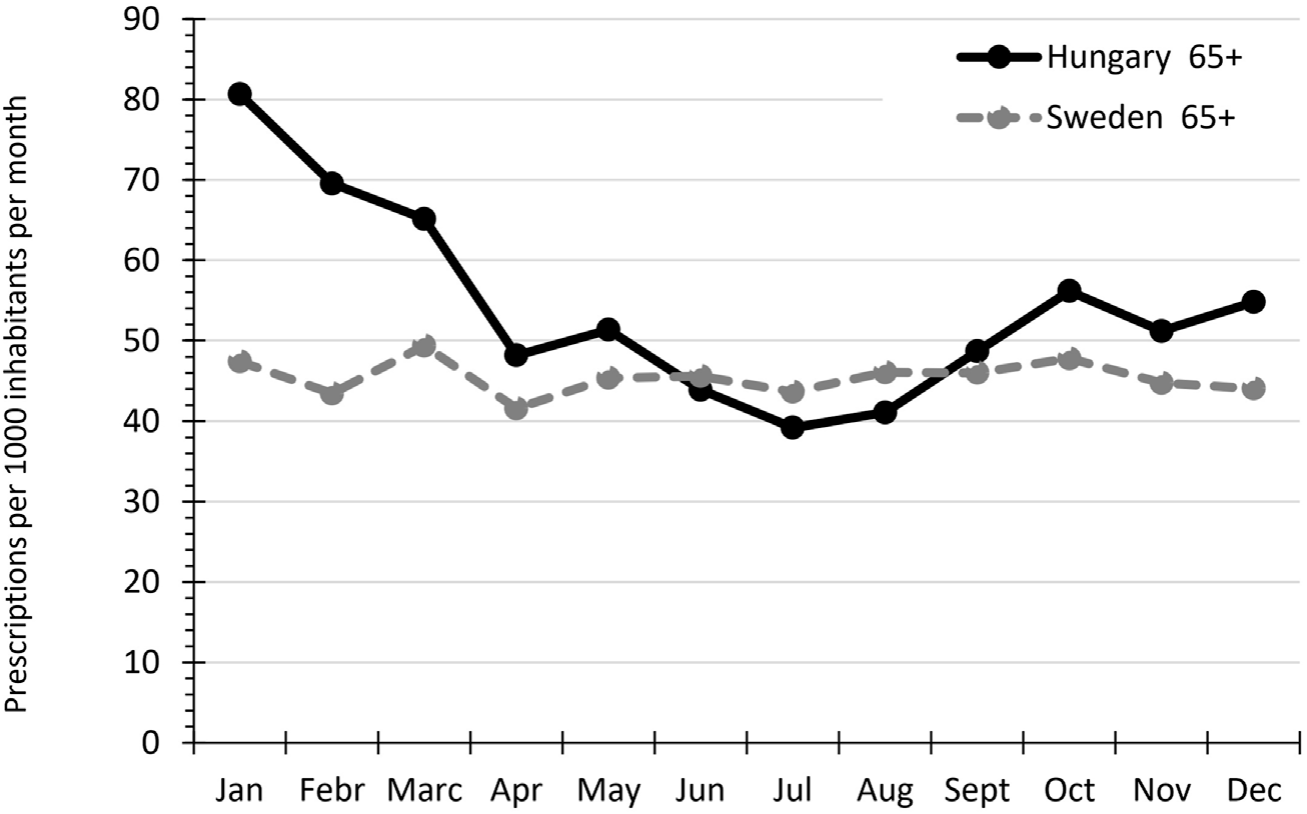
Seasonal variation of antibiotic use among the elderly population in Hungary and Sweden in 2017.

Figure 3 shows the seasonal variation in antibiotic use in the elderly in Hungary and Sweden. The seasonal fluctuation was high in Hungary, reaching a peak of 80.7 prescriptions/1000 inhabitants/month in January. The lowest value in Hungary was 39.2 prescriptions/1000 inhabitants/month in July. Antibacterial use in the elderly population in Sweden was more equally distributed over the entire year, with a peak consumption of 49 prescriptions/1000 inhabitants/month in March and a nadir of 42 prescriptions/1000 inhabitants/month in April.

## Discussion

To the best of our knowledge, this is the first study to report on Hungarian data on antibiotic use in the elderly and the first age-specific comparison of antibiotic use between two countries. Our results showed that antibiotic exposure was higher in the Hungarian elderly population than in their Swedish counterparts. Several factors might explain the higher antibiotic exposure in the Hungarian elderly than in Sweden.

### Scale of use

Life expectancy is one of the most commonly used measures of the overall health of a population. The average life expectancy in 2017 for those aged 65 years was higher in Sweden than in Hungary (20.40 years vs 16.70 years), meaning that the Hungarian elderly has poorer health status (Eurostat, 2022).

Data on acute infection incidences are unavailable in the national statistics, but data on chronic disease prevalence, which can increase infection risk compared to the healthy population, is retrievable and can partly explain the observed differences between Hungary and Sweden. Two-thirds of Hungarians and nearly half of Swedish elderly (aged ≥65) reported at least one chronic disease (OECD, 2020). An epidemiological research revealed that patients with diabetes suffer infections more frequently than those without diabetes with consequent higher antibiotic use (Alves et al., 2012). The prevalence of diabetes in the elderly was higher in 2014 in Hungary than in Sweden (18.6% vs 12.6%) (Eurostat, 2014c). Obesity has also been an independent risk factor for infections in retrospective and prospective studies (Harpsøe et al., 2016). It increases the risk of pneumococcal respiratory tract infections (RTI), skin, gastrointestinal tract, and urinary tract infections (UTI) in elderly individuals (Frasca and McElhaney, 2019; Ghilotti et al., 2019). The prevalence of obesity in the elderly was much higher in 2014 in Hungary than in Sweden (26.5% vs 14.5%) (Eurostat, 2014a).

Smoking is one of the main risk factors for RTI, and the rate of daily smokers among the elderly was higher in Hungary (10.8%) than in Sweden (7.2 %), however this difference is much higher in the overall population (28 % vs. 7%, in 2020). In addition, smoking increases infection risk for digestive, reproductive, and other systems, which could lead to slightly higher antibiotic use in Hungarian elderly than in Swedish (Jiang et al., 2020). The annual number of hospital discharges due to malignant neoplasm of the respiratory tract (trachea, bronchus, and lung) in 2017 was also higher in Hungarian elderly (13,115 patients) than in its Swedish counterparts (4,966 patients) (Eurostat, 2017a). Prescribers may have a lower threshold for initiating antibiotic use in patients with cancer because antibiotics have positive side effects, such as cancer apoptosis promotion, cancer growth inhibition, and cancer metastasis prevention, e.g., lung cancer (Gao et al., 2020).

The population’s low health literacy and health-related knowledge can contribute to patients’ attitudes, beliefs, perceptions, and behaviors related to antibiotic use and can result in higher overall antibiotic use (Salm et al., 2018). The Eurobarometer public survey from 2018 revealed that the Hungarian public’s knowledge of antibiotics was worse than Swedish because only 37% of respondents gave entirely correct answers for all four antibiotic knowledge-related questions in Hungary, while 74% in Sweden (WHO, 2018).

The Eurostat statistics from 2017 revealed that the proportion of Hungarian elderly with >10 GP visits per year was 20.0% (65–74 years) and 29.5% (≥75 years), while this rate was only 3.7% (65–74 years) and 5.8% (≥75 years or more) in Sweden, suggesting that GP visits have a lower threshold in the Hungarian elderly population, which can contribute to higher antibiotic use (Tyrstrup et al., 2017). In addition, of the surveyed people in Hungary in the Eurobarometer study, 25% stated antibiotic prescription for sore throat and 17% for fever, while 9% for sore throat and 2% for fever in Sweden (European Commission, 2016). Data suggests that initiating antibiotic treatment is less judicious among Hungarian doctors although this data is based on patient recalls. Misleading advertising can be partly responsible for this. Over-the-counter dorithricin-containing lozenges, a local antibiotic, were heavily advertised on TV as a “throat saver antibiotic” in earlier years in Hungary, sending the incorrect message both to patients and doctors that antibiotics are required to relieve sore throats.

Physicians are primarily responsible for the decision to use antibiotics; thus, ensuring the optimal attitudes and knowledge that underlie their prescribing habits is a prerequisite for improving prescription quality (Gonzalez et al., 2015). A recent study revealed a 20% proportion of final-year medical students who want more education on prudent antibiotic use in Sweden, while >71% in Hungary. This means that medical students in Sweden feel prepared for prudent antibiotic prescription in much higher percentages than final-year students in Hungary (Dyar et al., 2018).

Moreover, antibiotic use is influenced by the existence of a national antibiotic policy (WHO, 2011). Sweden implemented the WHO recommendations for antibiotic stewardship in the form of a national strategic program to combat antibiotic resistance (Medical Products Agency and Strama, 2008), which is a continuously evolving collaboration that has been in place since 1995 (Mölstad et al., 2017). In contrast, a national antibiotic policy is not implemented with clear targets, responsibilities, and dedicated funding in Hungary (WHO, 2018).

Market forces and manufacturers’ marketing activity can also largely influence prescription practices in Hungary (WHO, 2018). The number of generics is very high in Hungary because they aim to reduce the price as much as possible (MacKenzie et al., 2006; Wouters et al., 2017), which might promote higher antibiotic use.

Overall, our study revealed that elderly females were prescribed more antibiotics than males in both countries. This can be partly explained by the sex differences in GPs visiting rates, wherein the rate of Hungarian elderly with >10 GP visits per year was 17.7% and 28.6% for males aged 65–74 years and >75 years, respectively, while 21.5% and 30.0% in the same age groups for females.

The sex gap in antibiotic prescription can partly be explained by consultation behavior differences (Smith et al., 2018). Males and females communicate differently with healthcare professionals, and prescribers may have gender biases that affect their willingness to prescribe antibiotics, resulting in higher antibiotic use in females (Smith et al., 2018). Males in the oldest two age groups were prescribed more antibiotics in Hungary due to the higher prevalence of risk factors among males, such as smoking and excessive alcohol consumption (WHO, 2018). The number of elderly male smokers is double compared to elderly female smokers aged 65–74 years and is five times higher in >75 years old in Hungary. Meanwhile, both sexes are equally smokers in each age subgroup in Sweden (Eurostat, 2014).

#### Pattern of use

We found that the absolute and relative ambulatory care use of different antibacterial subgroups differed greatly in the elderly population between Hungary and Sweden. In Hungary, penicillin beta-lactamase combinations, such as co-amoxiclav were preferred, compared to Sweden where it was marginally used (19.08% vs 1.83%). The high use of co-amoxiclav has been estabilished in previous research as a drug of choice for RTI in Hungary (Matuz et al., 2013). Swedish policy recommends prescribing narrow-spectrum penicillins in ambulatory care for RTI (Aspevall et al., 2020) and our data indirectly indicate good adherence to this guideline. Surveillance report from the European Antimicrobial Resistance Surveillance Network (EARS-Net) showed that percentages of penicillin-resistant pneumococci (PRP) were similar in Hungary (6.9%) and Sweden (6.1%) (ECDC, 2017). Clavulanic acid use is not necessary for PRP because the resistance mechanism is not connected to the bacteria’s capability to produce beta-lactamase enzymes; hence, the addition of clavulanic acid to aminopenicillin will not help to overcome this resistance (Huttner et al., 2020). Co-amoxiclav is dominantly used compared to amoxicillin alone in Hungary because co-amoxiclav was placed on the market earlier than amoxicillin alone; thus, doctors became used to it (Benko, 2016). The use of broad-spectrum antibiotics, such as co-amoxiclav can compromise the host microbiome. Even short-term antibiotic exposure alters the gut microbiota and bacterial diversity recover after weeks or months after (Elvers et al., 2020). Disruption of the human microbiom by antibiotic use can lead to AMR infections and several diseases such as allergia, asthma, obesity or vitamin K deficiency (Langdon et al., 2016).

Quinolone was also more frequently used in Hungary than in Sweden (34.53% vs. 9.98% of total ambulatory use in the elderly, respectively). Previous research showed that fluoroquinolones were commonly used in ambulatory care to treat urinary tract infections and also RTIs in Hungary (Juhasz et al., 2013; Matuz et al., 2015; Benkő et al., 2020). Contrarily, pivmecillinam and nitrofurantoin were proved to be the first-line antibiotics to treat community-acquired UTIs in Sweden (Kornfält et al., 2019). The consequences of high fluoroquinolone use can be various. The Food and Drug Administration has placed a boxed warning on fluoroquinolone antibiotics which highlights older adults as being at an elevated risk of serious side effects, including tendon rupture, delirium, peripheral neuropathy, blood sugar disturbances, and aortic dissection (U.S. Food and Drug Administration, 2018). Fluoroquinolones also increase the risk of CDI (*Clostridioides difficile* infection) (Kabbani et al., 2018). Fluoroquinolone can cause QT interval prolongation and subsequently increase the risk of *torsades de pointes (TdP) type arrhythmias.* Given that heart failure and other risk factors such as uncorrected hypokalaemia, hypomagnesaemia might be present more frequently in the elderly, they are more vulnerable to potentially fatal cardiac arrhythmias such as TdP (Stahlmann & Lode, 2010). The 2017 annual report of the EARS-Net showed a difference in the percentage of fluoroquinolone-resistant *Escherichia coli* between Hungary and Sweden (30.6% vs 15.8%, respectively) that could be due to differences in the quinolone use in the two countries (ECDC, 2017).

The results of this comparison between the two countries are essential for Hungary since they need to optimize antibiotic use in the elderly to prevent serious adverse effects, more rapid resistance development, and higher costs (WHO, 2018). The availability of therapeutic guidelines might contribute to the observed pattern of antibiotic use in both countries. Up-to-date diagnostic and treatment guidelines have been unavailable for most community-associated infections for several years in Hungary, but Sweden continuously updates the guidelines every 3 years (Government Offices of Sweden, 2020).

#### Seasonal antibiotic use

The Hungarian antibiotic use in the elderly was very similar to Sweden in the summer months, but we detected substantially higher antibiotic use in the Hungarian elderly in the winter months. Seasonal fluctuation of outpatient antibiotic use in the general population across European countries has been previously described (Elseviers et al., 2007) and linked to an increased prevalence of RTI during the winter months, resulting in higher antibiotic prescription rates during this time (Elseviers et al., 2007).

Viral RTI and influenza-like syndromes were the most frequent infections in winter in both countries (Folkhälsomyndigheten, 2017; Kovács and Pakot, 2020); thus, antibiotics were possibly prescribed for self-limiting viral infections. The close correlation between viral respiratory infections, such as influenza and antibiotic prescriptions (Ryu et al., 2018), suggests that reducing the incidence of influenza through vaccination efforts in elderly people (Smetana et al., 2018) could help decrease the overprescription of antibiotics. The Eurostat in 2017 reported that Sweden has a higher vaccination rate against influenza in the population aged ≥65 years (49.8%) than in Hungary (26.8%) (Eurostat, 2017b), which might result in lower influenza illness rates in Sweden.

### Study strengths and limitations

The strength of this study is the nearly 100% population and drug coverage in both countries. However, some limitations need to be acknowledged. Firstly, this research only uses 1-year data from the two countries, which precludes analysis of annual trends in antibiotic use. Secondly, data is not stratified by specific indications. However, these limitations do not affect our aims and conclusions. Finally, we have to highlight, that systemic antibiotic use (WHO: J01) includes methenamine (urinary disinfectant) with considerable use in Sweden (sixth place on the top list). Excluding methenamine would result in even higher differences in the antibiotic utilization of the two countries.

## Conclusion

The scale and pattern of elderly ambulatory antibiotic use differed between Hungary and Sweden. Some of the observed differences could be explained by the different health statuses between the two populations; however, data suggest that interventions are needed to optimize antibiotic use in the elderly in Hungary.

## Data Availability

The data analyzed in this study is subject to the following licenses/restrictions: Aggregated datasets (without age, gender) is publicly available, while more detailed should be requested from the National Health Fund (NEAK). Requests to access these datasets should be directed to www.neak.gov.hu.

## References

[B1] Alves C. Casqueiro J. Casqueiro J. (2012). Infections in patients with diabetes mellitus: A review of pathogenesis. Indian J. Endocrinol. Metab. 16 (7), 27–S36. 10.4103/2230-8210.94253 PMC3354930 22701840

[B2] Aspevall O. Bergfeldt V. Nilsson O. Pringle M. (2020). Swedres | svarm 2017. Public Health Agency of Sweden and National Veterinary Institute. Shewa, Ethiopia 9 (9), 624. http://files/1182/Unknown - 2017 - 2017 Swedres Svarm.pdf%0Ahttp://files/522/Unknown - 2017 - 2017 Swedres Svarm.pdf.

[B3] Benkő R. Gajdács M. Matuz M. Bodó G. Lázár A. Hajdú E. (2020). Prevalence and antibiotic resistance of eskape pathogens isolated in the emergency department of a tertiary care teaching hospital in Hungary: A 5-year retrospective survey. Antibiotics 9 (9), 6244–E717. 10.3390/antibiotics9090624 PMC7560131 32961770

[B4] Benko R. Matuz M. Doro P. Hajdu E. Nagy G. Nagy E. (2016). [Antibiotic consumption between 1996 and 2003: National survey and international comparison] Orv. Hetil. 157, 1215–1222. 16898083

[B5] Blix H. S. Engeland A. Litleskare I. Rønning M. (2007). Age- and gender-specific antibacterial prescribing in Norway. J. Antimicrob. Chemother. 59 (5), 971–976. 10.1093/jac/dkm032 17329270

[B6] Dyar O. J. Nathwani D. Monnet D. L. Gyssens I. C. Lundborg C. S. Pulcini C. (2018). Do medical students feel prepared to prescribe antibiotics responsibly? Results from a cross-sectional survey in 29 European countries. J. Antimicrob. Chemother. 73 (8), 2236–2242. 10.1093/jac/dky150 29746647

[B7] ECDC (2017). “Ecdc: SURVEILLANCE REPORT. Annual report of the European antimicrobial resistance surveillance Network (EARS-Net) 2017,” in Surveillance of antimicrobial resistance in Europe. https://ecdc.europa.eu/en/publications-data/antimicrobial-resistance-surveillance-europe-2016.

[B8] Elseviers M. M. Ferech M. Vander Stichele R. H. Goossens H. Mittermayer H. Metz S. (2007). Antibiotic use in ambulatory care in Europe (ESAC data 1997-2002): Trends, regional differences and seasonal fluctuations. Pharmacoepidemiol. Drug Saf. 16 (1), 115–123. 10.1002/pds.1244 16700079

[B9] Elvers K. T. Wilson V. J. Hammond A. Duncan L. Huntley A. L. Hay A. D. (2020). Antibiotic-induced changes in the human gut microbiota for the most commonly prescribed antibiotics in primary care in the UK: A systematic review. BMJ Open 10 (9), e035677 10.1136/bmjopen-2019-035677 PMC7507860 32958481

[B10] European Commission (2016). “Special eurobarometer 445 report antimicrobial resistance,” in *Antimicrobial resistance* (issue April). http://ec.europa.eu/COMMFrontOffice/PublicOpinion.

[B11] European Union O, and OECD (2020). Health at a glance: Europe 2020: State of health in the EU cycle. PublishingOECD Publishing. Paris, France. 10.1787/9789264105133-ko

[B12] Eurostat (2014b). *Data-Eurostat. Daily smokers of cigarettes by sex, age and educational attainment level*. Eurostat https://ec.europa.eu/eurostat/databrowser/view/HLTH_EHIS_SK3E__custom_3202364/default/table?lang=en.

[B13] Eurostat (2017a). *Data-Eurostat. Hospital discharges by diagnosis and NUTS 2 regions, in-patients, per 100 000 inhabitants*. Eurostat https://ec.europa.eu/eurostat/databrowser/view/HLTH_CO_DISCH2T__custom_3202411/default/table?lang=en.

[B14] Eurostat (2017b). *Data-Eurostat. Vaccination against influenza of population aged 65 and over[hlth_ps_immu]*. Eurostat https://appsso.eurostat.ec.europa.eu/nui/submitViewTableAction.do.

[B15] Eurostat (2022). *Data-Eurostat. Life Expectancy at 65*. Eurostat https://ec.europa.eu/eurostat/statistics-explained/index.php?title=File:Table2_Life_expectancy_at_65.png.

[B16] Eurostat (2019). “Data - Eurostat,” in Eurostat. https://ec.europa.eu/eurostat/web.

[B17] Eurostat (2014d). Data-eurostat. https://ec.europa.eu/eurostat/web/.

[B18] Eurostat (2014a). Data-Eurostat. Body mass index (BMI) by sex, age and educational attainment level. Eurostat https://ec.europa.eu/eurostat/databrowser/view/HLTH_EHIS_BM1E__custom_3202342/default/table?lang=en.

[B19] Eurostat (2014c). Eurostat. http://appsso.eurostat.ec.europa.eu/nui/submitViewTableAction.do.Data-Eurostat. Persons reporting a chronic disease, by disease, sex, age and educational attainment level

[B20] Feehan J. Tripodi N. Apostolopoulos V. (2021). The twilight of the immune system: The impact of immunosenescence in aging. Maturitas 147, 7–13. 10.1016/j.maturitas.2021.02.006 33832647

[B21] Folkhälsomyndigheten (2017). Influenza in Sweden 2016-2017 season. http://www.folkhalsomyndigheten.se/publicerat-material/publikationer/.

[B22] Frasca D. McElhaney J. (2019). Influence of obesity on pneumococcus infection risk in the elderly. Front. Endocrinol. 10 (FEB), 71–78. 10.3389/fendo.2019.00071 PMC6381016 30814978

[B23] Gao Y. Shang Q. Li W. Guo W. Stojadinovic A. Mannion C. (2020). Antibiotics for cancer treatment: A double-edged sword. J. Cancer 11 (17), 5135–5149. 10.7150/jca.47470 32742461 PMC7378927

[B24] Ghilotti F. Bellocco R. Ye W. Adami H. O. Trolle Lagerros Y. (2019). Obesity and risk of infections: Results from men and women in the Swedish national March cohort. Int. J. Epidemiol. 48 (6), 1783–1794. 10.1093/ije/dyz129 31292615

[B25] Gonzalez-Gonzalez C. López-Vázquez P. Vázquez-Lago J. M. Piñeiro-Lamas M. Herdeiro M. T. Arzamendi P. C. (2015). Effect of physicians’ attitudes and knowledge on the quality of antibiotic prescription: A cohort study. PLoS ONE 10 (10), e0141820. 10.1371/journal.pone.0141820 26509966 PMC4624842

[B26] Government Offices of Sweden (2020). Swedish strategy to combat antibiotic resistance 2020-2023. https://www.government.se/499178/globalassets/government/dokument/socialdepartementet/amr_strategi_eng_web.pdf.

[B27] Harpsøe M. C. Nielsen N. M. Friis-Møller N. Andersson M. Wohlfahrt J. Linneberg A. (2016). Body mass index and risk of infections among women in the Danish national birth cohort. Am. J. Epidemiol. 183 (11), 1008–1017. 10.1093/aje/kwv300 27188940

[B28] Huttner A. Bielicki J. Clements M. N. Frimodt-Møller N. Muller A. E. Paccaud J. P. (2020). Oral amoxicillin and amoxicillin–clavulanic acid: Properties, indications and usage. Clin. Microbiol. Infect. 26 (7), 871–879. 10.1016/j.cmi.2019.11.028 31811919

[B29] Jensen M. L. V. Aabenhus R. M. Holzknecht B. J. Bjerrum L. Jensen J. N. Siersma V. (2021). Antibiotic prescribing in Danish general practice in the elderly population from 2010 to 2017. Scand. J. Prim. Health Care 39 (4), 498–505. 10.1080/02813432.2021.2004754 34818137 PMC8725860

[B30] Jiang C. Chen Q. Xie M. (2020). Smoking increases the risk of infectious diseases: A narrative review. Tob. Induc. Dis. 18 (July), 60. 10.18332/tid/123845 32765200 PMC7398598

[B31] Juhasz Z. Benko R. Matuz M. Viola R. Soos G. Hajdu E. (2013). Treatment of acute cystitis in Hungary: Comparison with national guidelines and with disease-specific quality indicators. Scand. J. Infect. Dis. 45 (8), 612–615. 10.3109/00365548.2013.777157 23547569

[B32] Kabbani S. Palms D. Bartoces M. Stone N. Hicks L. A. (2018). Outpatient Antibiotic prescribing for older adults in the United States: 2011 to 2014. J. Am. Geriatr. Soc. 66 (10), 1998–2002. 10.1111/jgs.15518 30221746 PMC7909599

[B33] Kornfält Isberg H. Melander E. Hedin K. Mölstad S. Beckman A. (2019). Uncomplicated urinary tract infections in Swedish primary care; Etiology, resistance and treatment. BMC Infect. Dis. 19 (1), 155–158. 10.1186/s12879-019-3785-x 30760219 PMC6375206

[B34] Kovács K. Pakot L. (2020). [Influenza-associated mortality in Hungary between 2009/2010 and 2016/2017]. Orv. Hetil. 161 (23), 962–970. 10.1556/650.2020.31725 32453699

[B35] Langdon A. Crook N. Dantas G. (2016). The effects of antibiotics on the microbiome throughout development and alternative approaches for therapeutic modulation. Genome Med. 8 (1), 39. 10.1186/s13073-016-0294-z 27074706 PMC4831151

[B36] MacKenzie F. M. Monnet D. L. Gould I. M. (2006). Relationship between the number of different antibiotics used and the total use of antibiotics in European hospitals. J. Antimicrob. Chemother. 58 (3), 657–660. 10.1093/jac/dkl286 16854957

[B37] Matuz M. Benkő R. Hajdú E. Viola R. Soós G. (2013). [Evaluation of ambulatory antibiotic use in Hungary using drug-specific quality indicators]. Orv. Hetil. 154 (24), 947–956. 10.1556/OH.2013.29632 23752050

[B38] Matuz Maria Bognar J. Hajdu E. Doro P. Bor A. Viola R. (2015). Treatment of community-acquired pneumonia in adults: Analysis of the national dispensing database. Basic Clin. Pharmacol. Toxicol. 117 (5), 330–334. 10.1111/bcpt.12426 26046802

[B39] Medical Products Agency and Strama (2008). Management of Respiratory Tract Infections. Sweden. [In Swedish] https://www.lakemedelsverket.se/sv/behandling-och-forskrivning/behandlingsrekommendationer/sok-behandlingsrekommendationer/antibiotika-vid-nedre-luftvagsinfektioner-i-oppenvard---behandlingsrekommendation#hmainbody1.

[B40] Mölstad S. Löfmark S. Carlin K. Erntell M. Aspevall O. Blad L. (2017). Lessons learnt during 20 years of the Swedish strategic programme against antibiotic resistance. Bull. World Health Organ. 95 (11), 764–773. 10.2471/BLT.16.184374 29147057 PMC5677604

[B41] Raban M. Z. Gates P. J. Gasparini C. Westbrook J. I. (2021). Temporal and regional trends of antibiotic use in long-term aged care facilities across 39 countries, 1985-2019: Systematic review and meta-analysis. PloS One 16 (8). 10.1371/journal.pone.0256501 e0256501 PMC8382177 34424939

[B42] Ryu S. Kim S. Kim B. I. Klein E. Y. Yoon Y. K. Chun B. C. (2018). Temporal relationship between antibiotic use and respiratory virus activities in the republic of Korea: A time-series analysis. Antimicrob. Resist. Infect. Control 7, 56. 10.1186/s13756-018-0347-8 29736236 PMC5922305

[B43] Salm F. Ernsting C. Kuhlmey A. Kanzler M. Gastmeier P. Gellert P. (2018). Antibiotic use, knowledge and health literacy among the general population in Berlin, Germany and its surrounding rural areas. PLoS One 13 (2). 10.1371/journal.pone.0193336 e0193336 PMC5825110 29474470

[B44] Smetana J. Chlibek R. Shaw J. Splino M. Prymula R. (2018). Influenza vaccination in the elderly. Hum. Vaccin. Immunother. 14 (3), 540–549. 10.1080/21645515.2017.1343226 28708957 PMC5861798

[B45] Smith D. R. M. Dolk F. C. K. Smieszek T. Robotham J. V. Pouwels K. B. (2018). Understanding the gender gap in antibiotic prescribing: A cross-sectional analysis of English primary care. BMJ Open 8 (2), e020203–e020207. 10.1136/bmjopen-2017-020203 PMC5855331 29472269

[B46] Stahlmann R. Lode H. (2010). Safety considerations of fluoroquinolones in the elderly: An update. Drugs Aging 27 (3), 193–209. 10.2165/11531490-000000000-00000 20210367

[B47] Tyrstrup M. van der Velden A. Engstrom S. Goderis G. Molstad S. Verheij T. (2017). Antibiotic prescribing in relation to diagnoses and consultation rates in Belgium, The Netherlands and Sweden: Use of European quality indicators. Scand. J. Prim. Health Care 35 (1), 10–18. 10.1080/02813432.2017.1288680 28277045 PMC5361413

[B48] U.S. Food and Drug Administration (2018). Drug safety communication: FDA warns about increased risk of ruptures or tears in the aorta blood vessel with fluoroquinolone antibiotics in certain patients. U.S. Food and Drug Administration https://www.jwatch.org/na48248/2019/02/13/adverse-effects-fluoroquinolones-where-do-we-stand.

[B49] Ventola C. L. (2015) The antibiotic resistance crisis: Part 1: Causes and threats P Trans. 40 (40), 277–283. 10.1016/B978-1-4831-9711-1.50022-3 PMC4378521 25859123

[B50] Who (2018). Evidence brief for policy: promoting the appropriate use of antibiotics to contain antibiotic resistance in human medicine in Hungary, 2. Evidence Informed Policy Network (EVIPNet) Europe.

[B51] Who (2020). ATC/DDD index 2022. WHO Publications. https://www.whocc.no/atc_ddd_index/.

[B52] Who (2011). European strategic action plan on antibiotic resistance. http://www.euro.who.int/__data/assets/pdf_file/0008/147734/wd14E_AntibioticResistance_111380.pdf.6September12–15.

[B53] Wouters O. J. Kanavos P. G. Mckee M. A. R. T. I. N. (2017). Comparing generic drug markets in Europe and the United States: Prices, volumes, and spending. Milbank Q. 95 (3), 554–601. 10.1111/1468-0009.12279 28895227 PMC5594322

